# Sustainability of High-Level Isolation Capabilities among US Ebola Treatment Centers

**DOI:** 10.3201/eid2306.170062

**Published:** 2017-06

**Authors:** Jocelyn J. Herstein, Paul D. Biddinger, Shawn G. Gibbs, Aurora B. Le, Katelyn C. Jelden, Angela L. Hewlett, John J. Lowe

**Affiliations:** University of Nebraska Medical Center, Omaha, Nebraska, USA (J.J. Herstein, K.C. Jelden, A.L. Hewlett, J.J. Lowe);; Harvard Medical School, Boston, Massachusetts, USA (P.D. Biddinger);; Indiana University School of Public Health, Bloomington, Indiana, USA (S.G. Gibbs, A.B. Le)

**Keywords:** Ebolavirus, Ebola virus disease, viruses, communicable diseases, hospitals, isolation, United States, Ebola

## Abstract

To identify barriers to maintaining and applying capabilities of US high-level isolation units (HLIUs) used during the Ebola virus disease outbreak, during 2016 we surveyed HLIUs. HLIUs identified sustainability challenges and reported the highly infectious diseases they would treat. HLIUs expended substantial resources in development but must strategize models of sustainability to maintain readiness.

During the 2014–2016 West Africa Ebola virus disease (EVD) outbreak, 56 hospitals in the United States were designated by the Centers for Disease Control and Prevention as Ebola treatment centers (ETCs). ETCs added national capacity to care for patients with highly infectious diseases (HIDs); that is, hazardous, easily transmissible, life-threatening illnesses with limited treatment options, such as EVD and severe acute respiratory syndrome coronavirus (*1*). ETCs were equipped with the clinical care resources, specialized infrastructure, and trained staff to safely manage and treat a person suspected or confirmed to have EVD (*2*). After the initial designation, 1 ETC in each US Department of Health and Human Services region was selected as a Regional Ebola and Other Special Pathogens Treatment Center (RESPTC) capable of managing HIDs for extended periods (*3*).

In 2009, a consensus group of infectious disease experts in Europe defined high-level isolation units (HLIUs) as facilities providing optimal infection containment and procedures specifically designed for HID care and released specifications for such units (*1*). A 2015 pilot survey of US HLIUs described the actions taken to establish high-level isolation capabilities and identified the costs of those efforts (*4*–*6*). The survey revealed that 45 of the US hospitals spent a cumulative total of $53.9 million (nearly $1.2 million per facility) to stand up their specialized isolation units (*4*).

Because of the substantial expenses and operational challenges of maintaining readiness, how HLIUs can continue these efforts has been questioned (*7*). The EVD outbreak revealed vulnerabilities within the US healthcare and public health infrastructure to address HIDs. We aimed to identify barriers to maintenance of recently developed isolation and care capabilities, how those capabilities might be applied to outbreaks other than EVD, and further infrastructure and resources HLIUs would add if additional funding were available.

## The Study

In early 2016, we sent a 70-question survey to the original 56 designated US HLIUs, including the 10 RESPTCs. The survey queried challenges and concerns about the maintenance of capabilities. Results were collected through Adobe Acrobat Pro (https://acrobat.adobe.com/us/en/acrobat/acrobat-pro.html) and analyzed by using descriptive statistics. The University of Nebraska Medical Center Institutional Review Board declared the study exempt (#172–16X).

Thirty-six (64%) hospitals responded. Of the 33 that completed the full survey, 3 reported they no longer maintained their HLIU capabilities. The 2 that provided qualitative information about their decision to close reported needing HLIU resources for other, more pressing areas and cited close proximity to at least 1 other HLIU as reasons for closing.

Nineteen (58%) hospitals reported using their HLIU for non-HID patients when not activated; the other 14 (42%) use the unit exclusively for patients with HIDs or for training (Table 1). When the 19 hospitals with adaptive isolation units (i.e., units otherwise used for normal hospital care) are activated, an average of 6.31 beds (median 6, range 2–12) must be taken offline when caring for 1 patient with an HID and an average of 6.97 beds (median 7.75, range 2–12) for 2 patients. Ten (53%) HLIUs with adaptive units stated preference for a unit dedicated to care for patients with HIDs; however, when asked the estimated costs of developing a unit for 2 HID patients, estimates ranged from $1 million to $12 million. Perceived benefits of a dedicated unit included minimizing disruption of other patients (4 hospitals), a constant state of readiness (3 hospitals), and an ability to train in the unit (4 hospitals).

**Table 1:** Activation of HLIUs and management of PUIs, United States*

Variable	Facilities, no./total no. (%)
Activation of HLIU	
HLIU can be activated 24/7 throughout the year†	32/33 (97)
Standing protocol exists to contact team members 24/7	31/33 (94)
Involve local/state public health officials in managing public concerns	32/33 (97)
PUIs	
Plan to provide care for PUIs and persons with confirmed cases	32/33 (97)
Staff used to care for PUI	
Use only HLIU staff to care for a PUI	28/32 (88)
Use other staff before disease is confirmed	4/32 (13)
Placement of PUI	
Place PUI exclusively in the HLIU while being assessed	14/32 (44)
Place PUI in either HLIU or hospital ED	12/32 (38)
Place PUI in ED until confirmed diagnosis	4/32 (13)
Other‡	2/32 (6)

*ED, emergency department; HLIU, high-level isolation unit; PUI, patient under investigation. †Average time necessary to activate HLIU after notification of pending patient transfer is 4.58 h (median 4 h, range 1.24 h). ‡One facility sends a mobile response team to a PUI’s home for evaluation, and another plans to use a mobile treatment unit (i.e., tent) for PUI placement.

Our initial 2015 survey reported that hospitals designated as ETCs incurred an average per hospital of $1,197,993 (*4*). Since that time, 25 (76%) of those original facilities reported receiving some degree of federal reimbursement, and 8 (24%) had not received any reimbursement. A cumulative total of $28,146,558 in federal funding (average $1,407,328, range $33,650–$6,000,000) was reported by the 20 (60%) reporting HLIUs. After we excluded federally funded RESPTCs and HLIUs that did not report initial investments in the pilot survey, the remaining 14 HLIUs reported a gap in reimbursement of $9,113,072.50 (mean $650,933.75 per HLIU).

**Figure 1:**
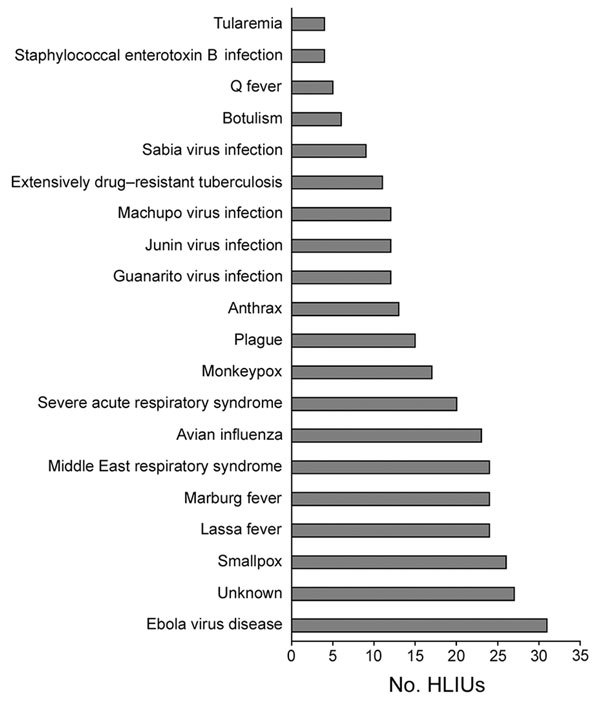
Diseases that 31 HLIUs reported they would treat, United States, 2016. HLIU, high-level isolation unit.

**Figure 2:**
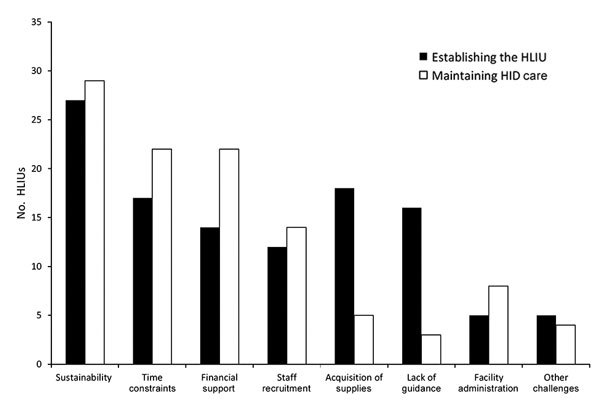
Challenges to establishing an HLIU and to maintaining HID care reported by survey respondents, United States, 2016 (n = 32 HLIUs). Other challenges include external support, lack of dedicated unit space, competing priorities, staffing needs, and decreasing hospital capacities. HLIU, high-level isolation unit; HID, highly infectious disease.

Although 1 HLIU reported lacking specific protocols or an ability to care for patients with an HID other than EVD, all other HLIUs (97%) reported being prepared to care for patients with HIDs other than EVD (Figure 1). Our survey also queried HLIUs about the challenges they experienced and challenges they foresee in maintaining the capabilities and capacity needed for HID care (Figure 2). Sustainability concerns was the most cited challenge in establishing and maintaining a HLIU. HLIUs also detailed facility modifications and/or capabilities they would add if additional hypothetical funding were available (Table 2).

**Table 2:** Operational capabilities HLIUs reported they would add or construct if funding were available, United States*

Funding amount	Capability	No. HLIUs
$100,000	Additional training/drills (e.g., for other diseases, purchase of simulation equipment)	6
	Broad supplies/equipment (e.g., beds, ventilators, family support technology/equipment)	4
	Laboratory capability and capacity (e.g., reduced transport of materials, lab hood in unit, purchase of new decontamination equipment)	4
$500,000	On-site waste disposal	4
	Expanded and updated patient rooms	3
	Enhanced laboratory capabilities (e.g., additional laboratory tests, larger lab space)	3
	Expanded isolation unit (e.g., increase capacity of negative-pressure rooms)	2
$1,000,000	Renovated/expanded isolation unit	4
	Separate, permanent isolation unit	3
	Expanded training (e.g., increased frequency)	2

*Individual HLIUs self-reported data through an electronically administered survey administered in 2016. HLIU, high-level isolation unit.

## Conclusions

Developed during the height of the West Africa Ebola outbreak, most newly established US HLIUs invested immense resources and effort into preparing for patients with EVD. However, no formal network of HLIUs has been established in the United States, except for the 10 RESPTCs, and at least 3 former HLIUs no longer maintain HLIU capabilities. Moreover, 14 HLIUs not designated as RESPTCs reported having spent $9.1 million more than they have been reimbursed to initially develop HLIU capabilities. As a result, these hospitals reported struggling to fund ongoing operations and sustain readiness.

Although many facilities have created adaptable-use HLIUs because they lack the capital funds, space, or both to create a dedicated unit, such units have major disadvantages because healthcare workers are unable to train in the unit, existing patients must be relocated when the unit is activated for an HID patient, and multiple additional rooms must be taken off-line for the care of 1 patient with an HID (*8*). Thus, more than half of US HLIUs that routinely care for non-HID patients would build an HID-dedicated unit if funds were available. However, because future funding sources for non-RESPTCs are unclear, lessons on sustainability might be learned from flexible-use HLIUs in Italy and the Netherlands, which offer levels of containment based on a patient’s condition and offset costs by routine use (*1*).

Our study had several limitations. The data were self-reported and not validated by external sources. The current status of HLIUs that did not participate in the follow-up survey is unknown. A decrease in participation from the initial survey to the follow-up could also be due to the longer, more detailed follow-up and could indicate the lack of attention to this area now that the EVD outbreak is over. The study population was based solely on a list published by the Centers for Disease Control and Prevention (*9*) and does not include data from other hospitals that similarly tried to strengthen their ability to treat HID patients.

In conclusion, a network of hospitals capable of treating patients with HIDs was rapidly constructed in response to the recent EVD outbreak. However, without the impending threat of EVD or another HID on the immediate horizon, public attention on HID preparedness tends to waver, and governments tend to prioritize and shift funding elsewhere. Additional external funding sources remain generally uncertain for US HLIUs not designated as RESPTCs; therefore, these HLIUs must strategize methods and models of sustainability if they are to maintain capabilities and readiness.
